# Multifunctional Hydrogel with 3D Printability, Fluorescence, Biodegradability, and Biocompatibility for Biomedical Microrobots

**DOI:** 10.3390/molecules29143351

**Published:** 2024-07-17

**Authors:** Gang Wang, Sisi Wang, Tao Hu, Famin Shi

**Affiliations:** 1 School of Physics and Electronic Science, Guizhou Normal University, Guiyang 550025, China; 232100070252@gznu.edu.cn (S.W.);; 2 School of Integrated Circuit, Guizhou Normal University, Guiyang 550025, China

**Keywords:** multifunctional hydrogel, biomedical microrobots, 3D printable, fluorescence imaging, biodegradable, biocompatible

## Abstract

As micron-sized objects, mobile microrobots have shown significant potential for future biomedical applications, such as targeted drug delivery and minimally invasive surgery. However, to make these microrobots viable for clinical applications, several crucial aspects should be implemented, including customizability, motion-controllability, imageability, biodegradability, and biocompatibility. Developing materials to meet these requirements is of utmost importance. Here, a gelatin methacryloyl (GelMA) and (2-(4-vinylphenyl)ethene-1,1,2-triyl)tribenzene (TPEMA)-based multifunctional hydrogel with 3D printability, fluorescence imageability, biodegradability, and biocompatibility is demonstrated. By using 3D direct laser writing method, the hydrogel exhibits its versatility in the customization and fabrication of 3D microstructures. Spherical hydrogel microrobots were fabricated and decorated with magnetic nanoparticles on their surface to render them magnetically responsive, and have demonstrated excellent movement performance and motion controllability. The hydrogel microstructures also represented excellent drug loading/release capacity and degradability by using collagenase, along with stable fluorescence properties. Moreover, cytotoxicity assays showed that the hydrogel was non-toxic, as well as able to support cell attachment and growth, indicating excellent biocompatibility of the hydrogel. The developed multifunctional hydrogel exhibits great potential for biomedical microrobots that are integrated with customizability, 3D printability, motion controllability, drug delivery capacity, fluorescence imageability, degradability, and biocompatibility, thus being able to realize the real in vivo biomedical applications of microrobots.

## 1. Introduction

Microrobots are micron-sized devices that can be utilized in hard-to-access environments, particularly in blood vessels and extracellular matrixes within the human body [1,2]. Therefore, microrobots have shown significant potential for biomedical applications, such as targeted drug delivery [3,4,5], minimally invasive surgery [6,7,8], and sensing and diagnosis [9,10,11]. However, microrobots will encounter challenges when interacting with biological tissues, complex biofluidic environments, and when overlapping with multiple stimuli when applied in vivo [12]; hence, several major challenges should be considered to enable the real in vivo biomedical applications of microrobots.

Firstly, the microrobots should be customizable, since the complex multilevel in vivo environment requires functional microrobots with different structures and materials [13]. The fabrication methods of microrobots include self-scrolling [14], glancing angle deposition (GLAD) [15], template-assisted deposition [16,17], and 3D laser direct writing [18,19,20]. Among them, 3D laser direct writing is the most promising method to achieve the customizability of microrobots. Secondly, the microrobots should be motion-controllable to achieve precise operation in the body. Several actuate approaches such as magnetic [21,22,23,24,25], electric [26,27], chemical [28,29], temperature [30,31], light [32], and ultrasound [33,34] were researched. Among them, magnetic fields can permeate through organisms remotely and harmlessly to tissues in a wide range of frequencies and magnitudes, which makes magnetic actuation one of the most promising approaches for biomedical applications [35,36]. Thirdly, the microrobots used in vivo should be imageable to be located accurately and controlled precisely. To achieve this goal, various methods are being researched, including fluorescence imaging [37,38,39], magnetic resonance imaging (MRI) [40,41], and photoacoustic imaging [42]. Among them, fluorescence imaging is widely used in biomedical imaging of microrobots due to its low cost, harmlessness to living organisms, and high image resolution [43]. Fourthly, the microrobots should be biodegradable to minimize side effects, since the biodegradable microrobots can be degraded and excreted from the body after the operation. Finally, the microrobots should be biocompatible, which ensures that the microrobots will not be attacked by immune cells when used in vivo.

Since the real in vivo biomedical applications of microrobots need to be 3D printable, motion-controllable, imageable, biodegradable, and biocompatible, researchers are devoted to developing materials that can simultaneously meet these application conditions. In the early research of microrobots, researchers mainly focused on the customizability, motion controllability, and functionality of microrobots; thus, rigid materials were mainly used at this time, such as SU-8 photoresist, IPL photoresist, and natural materials [14,15,19]. Although the biocompatibility and imageability issues of rigid materials can be addressed by titanium coating and near-infrared molecules NIR-797 modifying the surface, respectively [37,44], the biodegradability of rigid materials remains unresolved. Therefore, researchers have turned their attention to soft materials. Among them, hydrogels are one of the most promising biodegradable and biocompatible soft materials for biomedical applications, and thus have been extensively researched. The hydrogels exhibit excellent feature of water absorption [45,46], which can be used for the delivery of water-soluble drugs. Degradable monomer such as GelMA can be employed to achieve the degradability of the hydrogel soft material; thus, the hydrogel can degrade into small molecules and be excreted from the body [47,48,49,50,51]. Furthermore, hydrogels are easier to achieve biocompatibility since their physicochemical properties are similar to those of tissues [52,53,54,55]. However, although there are many studies on hydrogel microrobots, few researchers can simultaneously fulfill the 3D printable, motion-controllable, imageable, biodegradable, and biocompatible requirements of microrobots, with only two or three being considered in most cases.

Herein, a multifunctional hydrogel was proposed to integrate the benefits of 3D printability, fluorescence imageability, biodegradability, and biocompatibility simultaneously. The hydrogel is composed of GelMA as the monomer, TPEMA as the fluorescent agent, poly(ethylene glycol) diacrylate (PEGDA) as the crosslinker, lithium phenyl-2,4,6-trimethylbenzoylphosphinate (LAP) as the photoinitiator, and triethanolamine (TEOA) as the photosensitizer. Based on the developed multifunctional hydrogel, a 3D-printed spherical microrobot coated with magnetite nanoparticles for drug delivery has been developed, which exhibits excellent movement performance. The swelling properties of the hydrogel suggested its great drug loading/release capacity. Fluorescence experiments have demonstrated the excellent fluorescence stability of hydrogel, which makes it suitable for use in biomedical fluorescence imaging applications. Moreover, the proposed hydrogel was verified to be degraded in 20 h by using collagenase. Finally, in vitro experiments using L929 cells were performed to confirm the biocompatibility of the hydrogel microstructure, which validated the non-toxicity, good cell adhesion, and growth of the hydrogel microstructure. The multifunctional hydrogel proposed here serves as the material proof-of-concept for future in vivo biomedical applications of microrobots.

## 2. Results and Discussion

### 2.1. Synthesis of Multifunctional Hydrogel

**Figure 1:**
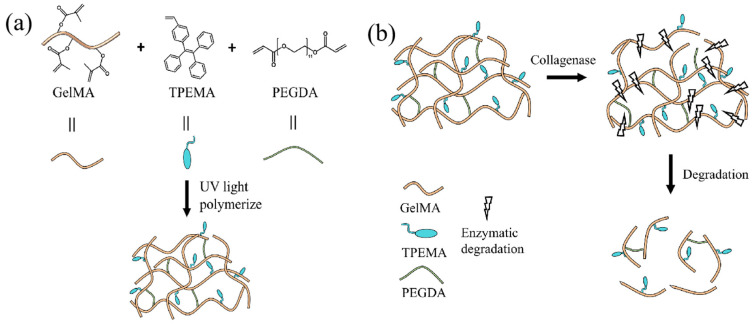
Illustration of polymerization and enzymatic degradation process of multifunctional hydrogel. (**a**) The main polymerization reaction involved in the 3D printing process. (**b**) The main degradation process of hydrogel using collagenase.

The precursor solution of multifunctional hydrogel that consists of GelMA, PEGDA, TPEMA, TEOA, LAP, and tetrahydrofuran (THF) was developed first. GelMA was utilized as the primary monomer due to its excellent biocompatibility and controllable mechanical properties, as well as GelMA hydrogels were proteolytically degradable by cell-secreted proteases in the extracellular matrix [56]. Therefore, GelMA has been extensively used in cell 3D culturing, tissue engineering, and biological 3D printing [57,58]. TPEMA was employed as the fluorescent agent, owing to its unique aggregation-induced luminescence effect, which has shown great application prospects in biological probes, photoelectric materials, and sensor materials [59,60,61]. PEGDA served as the crosslinker to form the network structure of the hydrogel. TEOA acted as the photosensitizer, which was used to polymerize polymer compounds during 3D laser direct writing. LAP was the photoinitiator, while THF was used as the transition solvent for dissolving the TPEMA, which helped to promote the photocuring process of hydrogel. The cross-linking process of the multifunctional hydrogel is illustrated in Figure 1a. During the printing process, the photoinitiator LAP was excited to produce radicals using the laser beam of the 3D DLW system with the assistance of the photosensitizer TEOA, and then the active free radicals underwent a polymerization reaction with the GelMA monomers, leading to chain elongation and the formation of linear polymers. Meanwhile, the crosslinker PEGDA was also activated using free radicals, and thereby led to cross-linking reactions between the formed polymers chains to interconnect and constitute the hydrogel framework. Furthermore, the copolymers between fluorescent agent TPEMA and GelMA monomers were also formed using free radical polymerization during the 3D printing process, indicating that the TPEMA fluorescent molecules were grafted onto GelMA monomers through chemical bonds to obtain the hydrogel with stable properties. As a result, the developed hydrogel demonstrated the excellent ability to absorb water and aqueous solutions, indicating its capacity to load water-soluble drugs [62]. 

Figure 1b depicts the enzymatic degradation process of the multifunctional hydrogel. Since the collagenase can break the polypeptide bonds within the gelatin molecules into small molecules, the skeleton of GelMA and TPEMA-based hydrogel can be broken by applying collagenase, thus degrading the hydrogel structures. With the enzymatic degradability and biocompatibility of GelMA, along with the fluorescence characteristics of TPEMA, the hydrogel introduced herein exhibits 3D printability, fluorescence imageability, biocompatibility, and degradability. These benefits enable the hydrogel microrobots to be applied in vivo for real medical applications.

### 2.2. Three-Dimensional-Printability and Microrobot Design

**Figure 2:**
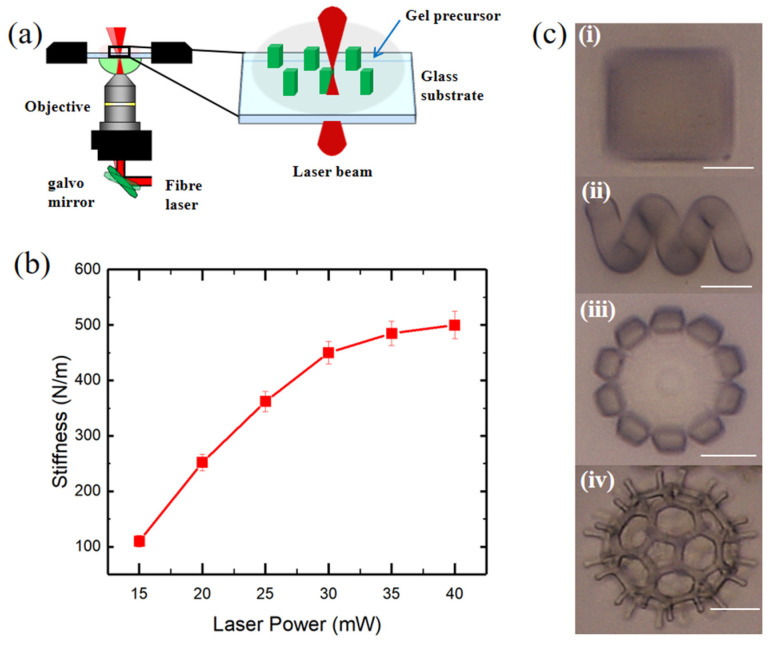
Illustration of the 3D printability of developed multifunctional hydrogel. (**a**) The schematic diagram of the 3D printing process of the hydrogel using a DLW system. A pulsed two-photon femtosecond fiber laser with an emission wavelength of 780 nm reaches the objective and results in photopolymerization. (**b**) The correlation between the mechanical properties (stiffness) of the printed hydrogel microstructures and the laser power. (**c**) Different structures designed and 3D-printed with developed hydrogel: (**i**) cube, (**ii**) helix, (**iii**) petaloid, and (**iv**) C_60_. Scale bar: 20 μm.

In this study, we use a 3D direct laser writing (DLW) system (Nanoscribe GmbH, Eggenstein-Leopoldshafen, Germany) to construct the hydrogel microstructures using two-photon polymerization (TPP). The sketch of TPP process of the hydrogel is illustrated in Figure 2a, in which a pulsed two-photon femtosecond fiber laser with an emission wavelength of 780 nm and the Galvo (layer by layer) scanning writing mode were used to construct the developed multifunctional hydrogel on a glass substrate. In the TPP process, the laser beam was focused on a specific location in a gel precursor solution to form a tiny polymerization area. When the energy of two photons was absorbed simultaneously, the GelMA monomers in the gel precursor underwent polymerization reactions, thus forming polymer chains. By moving the laser beam, 3D complex microstructures could be accurately printed. Furthermore, The 3D DLW system could vary the exposure dose of the hydrogel by changing the laser power and scanning speed so as to achieve the construction of hydrogel microstructures with different cross-linking densities.

Since the 3D printability of hydrogels depends mainly on the mechanical property of the material at the micron scale, we also characterized the stiffness of developed hydrogel via an in situ micromechanical compression test, and the stiffness of the printed cuboid samples as a function of the laser power varying from 15 to 40 mW is demonstrated in Figure 2b. The stiffness of the hydrogel samples gradually increases with the increasing of laser power and eventually reaches nearly 500 N/m at 40 mW, which suggests the formation of hydrogel framework with high crosslinking density, thus can support the construction of complex 3D hydrogel micromachines. As a result, the developed multifunctional hydrogel exhibits excellent customizability and 3D printability. Figure 2c demonstrates its versatility by showcasing designs such as the cube, helix, petaloid, and C_60_ microstructures, all of which were fabricated using the 3D direct laser writing (DLW) method. 

**Figure 3:**
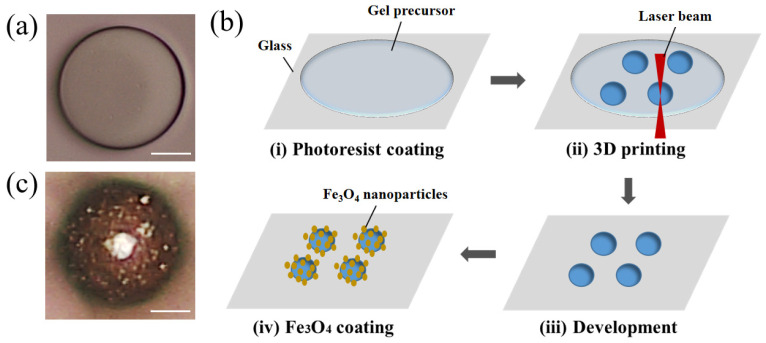
Illustration of hydrogel microrobots. (**a**) Optical image of microsphere. (**b**) Fabrication process of microspheres: (**i**) the developed hydrogel was dropped on a glass substrate with a pipette gun, (**ii**) microspheres were printed using the 3D direct laser writing (DLW) method, (**iii**) the sample was developed in deionized water, and (**iv**) the sample was coated with Fe_3_O_4_ nanoparticles. (**c**) Optical image of the microsphere with coated Fe_3_O_4_ nanoparticles. Scale bar: 20 μm.

As shown in Figure 3a, a spherical microrobot (i.e., microsphere) was designed to investigate the feasibility of the proposed hydrogel for biomedical applications, especially drug delivery. The spherical shape was chosen due to its low specific surface area, which reduces the risk of drug leakage during delivery. The fabrication process of microspheres is illustrated in Figure 3b. The developed multifunctional hydrogel was dropped on a glass substrate with a pipette gun, and microspheres were then printed using the 3D direct laser writing (DLW) method with a 780 nm wavelength femtosecond laser. The sample is then developed in deionized water and coated with Fe_3_O_4_ nanoparticles using ethyl lactate/Fe_3_O_4_ solution. The optical image of a hydrogel microsphere with coated Fe_3_O_4_ nanoparticles is shown in Figure 3c.

### 2.3. Swelling Properties

**Figure 4:**
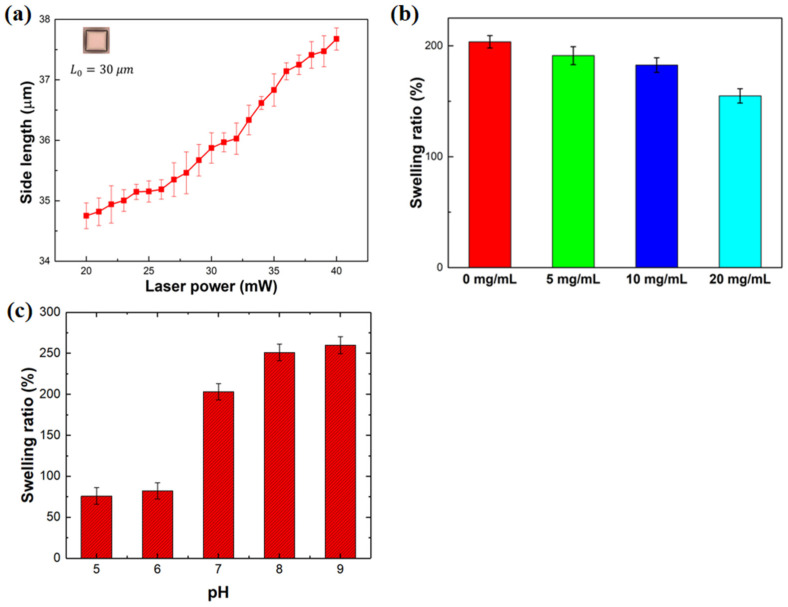
The swelling characteristics of the developed hydrogel. (**a**) Effect of laser power on the side length of the printed cuboid microstructures, and (**b**) the correlation between the swelling ratio of Fe_3_O_4_-coated cuboid microstructures and the concentration of ethyl lactate/Fe_3_O_4_ solution at pH = 7. (**c**) The effect of pH on swelling ratio of developed hydrogel cuboid microstructures.

The porous nature of hydrogels makes them capable of carrying large amounts of water, and thus they can be used as carriers of water-soluble drugs [63]. The drug-loading capacity of hydrogels depends on their swelling characteristics, which are determined by the crosslinking density of hydrogels. In the 3D printing process, the crosslinking density of hydrogels differs with exposure dosage when the hydrogel precursor is photocured. Therefore, to illustrate the effect of exposure dosage on swelling characteristics of the proposed hydrogel quantitatively, a series of cuboid samples with side lengths of 30 μm × 30 μm × 30 μm were created as the laser power gradually increases at a constant scanning speed of 8 mm/s. As demonstrated in Figure 4a, the side lengths of these samples at equilibrium states were plotted as a function of laser power at pH = 7. It was observed that an increase in the exposure dosage resulted in an corresponding increase in the side lengths of the cuboid samples. This is because the crosslinking density of the printed hydrogel increases with exposure dosage (i.e., laser power when scanning speed of 8 mm/s), which results in improved swelling performance. Therefore, the water absorption capacity of the hydrogel can be improved by appropriately increasing the printing laser power.

In addition, it is worth noting that the swelling characteristics of developed hydrogel are also related to the concentration of ethyl lactate/Fe_3_O_4_ solution coated on their surface. Figure 4b illustrates the correlation between the swelling ratio of Fe_3_O_4_-coated cuboid samples and the concentration of ethyl lactate/Fe_3_O_4_ solution (0 mg/mL, 5 mg/mL, 10 mg/mL, and 20 mg/mL) at pH = 7. The swelling ratio (κ) was defined as follows:

(1) $$\kappa (\%)=\frac{V-V_{0}}{V_{0}}\times 100\%,$$

where *V* and *V*_0_ are the swelling volume and initial dehydrated volume of Fe_3_O_4_-coated cuboid samples, respectively. The fabricated samples were under the condition of a scanning speed of 8 mm/s and a laser power of 23 mW, and thus had a initial dehydrated length of 24 μm. As is shown in Figure 4b, the swelling ratio decreases as the concentration of ethyl lactate/Fe_3_O_4_ solution increases, which owes to the fact that the Fe_3_O_4_ nanoparticles film deposited on the surface reduces the swelling area of Fe_3_O_4_-coated cuboid samples, as well as constraining the expansion of the cuboid samples. Moreover, the downward trend in Figure 4b declines faster with a higher concentration of ethyl lactate/Fe_3_O_4_ solution. Therefore, to enhance the water absorption capacity of hydrogel microrobots, the concentration of ethyl lactate/Fe_3_O_4_ solution should be appropriately reduced. 

Furthermore, the swelling ratio is also related to pH value of swelling media; thus, the effect of pH on swelling ratio of developed hydrogel cuboid microstructures was studied. As is shown in Figure 4c, the swelling ratio was strongly influenced by pH value, and when the external environment changes from acidic to neutral and then to alkaline, the swelling ratio increased dramatically. But when the developed hydrogel microstructures were in an acidic or alkaline condition, the effect of pH changes on its swelling ratio will be much smaller; as is demonstrated in Figure 4c, when the pH changed from 5 to 6 or 8 to 9, the swelling ratio increased slightly. Therefore, it could be concluded that the swelling performance of the developed hydrogel is affected by pH value of swelling medium, and it will be better in alkaline condition but worse in the acidic condition. 

### 2.4. Drug Loading and Release Capacity

**Figure 5:**
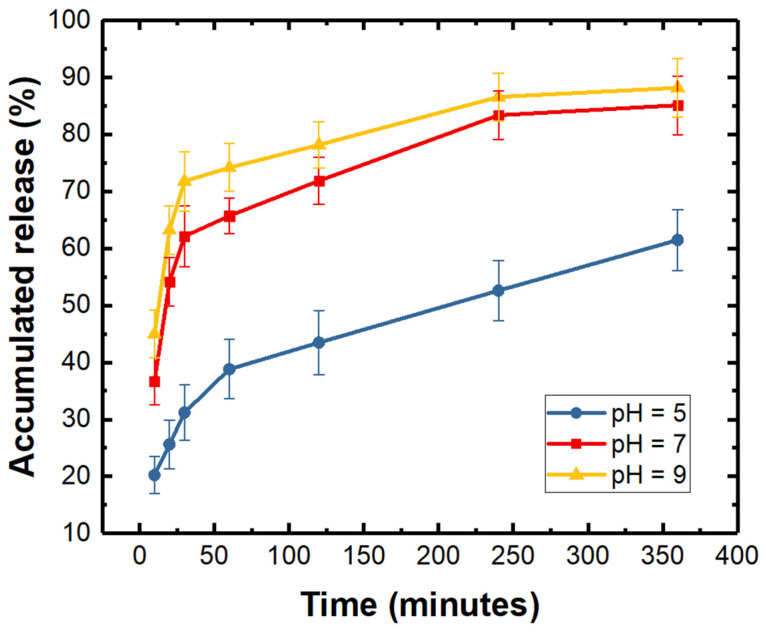
Accumulated release of urokinase from the cube-shaped hydrogel patch over time at different pH of release medium.

The developed hydrogel can be used for the delivery of water-soluble drugs due to its excellent water absorption capacity. Herein, to verify the drug loading and release capacity of the proposed hydrogel, we have conducted the drug release experiments in vitro. A 600 μm × 600 μm × 30 μm cube-shaped hydrogel patch was employed to conduct the in vitro drug release experiment because of its immobility and ease of operation [64,65,66]. Meanwhile, the urokinase was chosen as a model drug, since it is commonly used for the treatment of thrombus in the body, as well as its water-soluble, colorless, and transparent qualities after dissolving in saline. The hydrogel cube was first fully dried to eliminate water inside the hydrogel and then was immersed in the urokinase saline solution to fully absorb the urokinase drug molecules; then, the hydrogel cube filled with drugs was employed to proceed with the drug release experiments in PBS solutions. The PBS solution containing the released urokinase drug molecules was collected at intervals and analyzed for the accumulated release (defined as the percentage of the total amount of drug released at the current and previous time to the initial drug loading amount) of urokinase drug molecules using UV-Vis spectrophotometry, and the result is shown in Figure 5. It can be seen from this Figure that when pH = 7, the release rate of urokinase was initially fast within the first 2 h, and gradually slowed down thereafter. This is because the release of urokinase drugs is caused by diffusion. At the beginning, the concentration of drugs in the hydrogel differs greatly from the external environment, so the drug release faster. As the drug release process progresses, the concentration difference gradually decreases; thus, the drug release will gradually slow down. It is worth noting that the accumulated release of urokinase reached 85% after 6 h, indicating that the urokinase drug can be released within a few hours. Therefore, the developed hydrogel microrobots can be used for the loading and release of water-soluble drugs, and the drugs are expected to be released in a relatively short time. The result demonstrates great potential of the hydrogel microrobots for target drug delivery applications. 

Furthermore, the accumulated release of urokinase at different pHs of release medium (pH = 5, 7, 9) was performed. From Figure 5, we could find that when pH changed from 7 to 5, the release rates decreased dramatically; this may be due to the contraction of hydrogel in acidic condition, which hindered the diffusion of drugs from the hydrogel. In addition, when pH changed from 7 to 9, the release rates slightly increased, which was caused by the swelling of hydrogel. Therefore, it could be concluded that the pH value had great effect on the release behavior of the developed hydrogel, and the drug release capacity of developed hydrogel is better in alkaline conditions, but worse in acidic conditions.

### 2.5. Movement Performance of Microspheres

**Figure 6:**
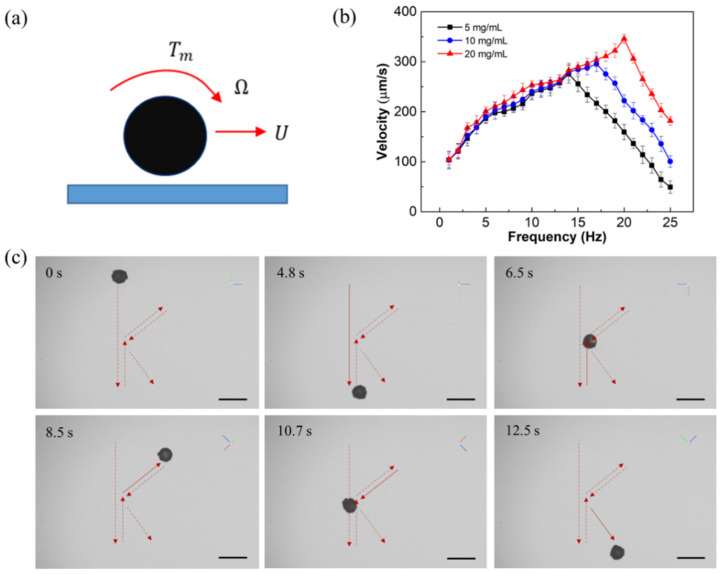
Movement performance of the developed hydrogel microspheres. (**a**) Schematic of movement of a microsphere on a plane. (**b**) Forward velocity of the microspheres with different Fe_3_O_4_ deposition concentrations. (**c**) Timelapse images of the microsphere with a controllable “K” shape at 14 mT and 1 Hz. Scale bars: 100 μm.

The motion controllability of microrobots is essential for biomedical applications; thus, the movement performance of the developed hydrogel microspheres was investigated. By depositing magnetic nanoparticles, the rotating magnetic field can be employed to actuate the hydrogel microspheres. As sketched in Figure 6a, a microsphere rotates on a plane with an angular velocity Ω and translates with a forward velocity U parallel to the plane under the action of the external magnetic moment $T_{m}$. As a result, a resistive force (*F*) and torque (*T*) from the fluid will be exerted on the microsphere, which can be expressed as follows [67]:

(2) $$F=6\pi \mu RF^{t*}U+6\pi \mu R^{2}F^{r*}\Omega ,$$

(3) $$T=8\pi \mu R^{2}T^{t*}U+8\pi \mu R^{3}T^{r*}\Omega ,$$

where μ is the fluid viscosity, *R* is the radius of the microsphere, $F^{t^{*}},F^{r^{*}}$ and $T^{t^{*}},T^{r^{*}}$ are the normalized scalar force and torque components, respectively. As is shown in Figure 3a,c, the surface of the 3D printed microsphere is smooth. But when the Fe_3_O_4_ nanoparticles are coated, the surface of the microsphere becomes rough due to the occurrence of clusters of nanoparticles. Since the fluid resistive force (*F*) and fluid torque (*T*) experienced by the microsphere with smooth surface and rough surface are different when moving on a smooth substrate, the movement of the Fe_3_O_4_-coated microsphere can be regarded as the motion of a micro-structured microsphere on a smooth substrate; thus, the normalized scalar force and torque components $F^{t^{*}},F^{r^{*}}$ and $T^{t^{*}},T^{r^{*}}$ can be expressed as follows [68]:

(4) $$F^{t^{*}}=(4/15)(\alpha +\beta )\ln(\delta /R)+O{\left(\delta /R\right)}^{0},$$

(5) $$F^{r^{*}}=(-2/15)\alpha \ln(\delta /R)+O{\left(\delta /R\right)}^{0},$$

(6) $$T^{t^{*}}=(-1/20)(\alpha +\beta )\ln(\delta /R)+O{\left(\delta /R\right)}^{0},$$

(7) $$T^{r^{*}}=(2/5)\alpha \ln(\delta /R)+O{\left(\delta /R\right)}^{0},$$

where the superscripts “*t*” and “*r*” represent the components of the translation and the rotation of the microsphere, respectively. δ is the gap between the plane and the bottom of the microsphere, and *R* is the radius of the microsphere. *α* and *β* represent the influence coefficient of the microstructure on the tangential velocity and radial velocity of the microsphere, respectively [68].

In addition, the external magnetic torque of the microsphere is given by the following:

(8) $$T_{m}=V\left|M\times B\right|=V\left|M\right|\left|B\right|\sin\theta ,$$

where V is the volume of magnetic material coated on the microsphere, **M** is the magnetization of the microsphere, **B** is the magnetic field vector, and θ is the angle between **M** and **B**, respectively. 

In our study, the hydrogel microsphere is only actuated by the external magnetic moment $T_{m}$; thus, the fluid force on the microsphere is  F=0 due to the force balance. Therefore, the uniform rolling of the hydrogel microsphere can be regarded as a series of equilibrium processes in the case of $T_{m}=T$, where T represents the fluid resistance torque. According to Equations (2) and (3), the relationship between  U and Ω and the step-out frequency $\Omega_{\mathrm{step}\text{-}\mathrm{out}}$ can be expressed as follows:

(9) $$\frac{U}{\Omega }=-\frac{RF^{r^{*}}}{F^{t^{*}}},$$

(10) $$\Omega_{\mathrm{step}\text{-}\mathrm{out}}=\frac{F^{t^{*}}}{8\pi \mu R^{3}\left(F^{t^{*}}T^{r^{*}}-F^{r^{*}}T^{t^{*}}\right)}T_{m}^{\max}.$$

When a microsphere moves on a substrate by applying a magnetic rotating field, it rolls steadily with a constant forward velocity, since the magnetic moment offsets the fluid resistance torque; thus, the rotational frequency of the microsphere synchronizes with the magnetic rotational frequency f. As a result, U shows an approximately linear relation against f when $f<f_{\mathrm{step}\text{-}\mathrm{out}}$, and then U and Ω reach the maximum when $f=f_{\mathrm{step}\text{-}\mathrm{out}}$. Thereafter, a further increase in f leads to the increasing in the fluid resistance torque while the magnetic moment remain unchanged, which results in a torque imbalance on the microsphere. Therefore, both the forward and the angular velocities of the microsphere will decrease to rebalance the resistance torque and the external magnetic moment. 

As is shown in Figure 6b, the forward velocities of hydrogel microspheres with different Fe_3_O_4_ deposition concentrations were almost the same when $f<f_{\mathrm{step}\text{-}\mathrm{out}}$, which is consistent with the theory derived from Equations (4), (5) and (9) that the different Fe_3_O_4_ deposition concentrations have little influence on the normalization coefficient $F^{t^{*}},F^{r^{*}}$. It can also be seen from Figure 6b that the step-out frequencies and maximum forward velocities of the hydrogel microspheres increased with increasing Fe_3_O_4_ deposition concentrations, which is also consistent with the theory derived from Equations (4)–(10) that the volume of magnetic material *V* increases with the increase in Fe_3_O_4_ deposition concentration, thereby increasing the step-out frequency and maximum forward velocity. Accordingly, it is possible to increase the Fe_3_O_4_ deposition concentration appropriately to achieve a larger forward velocity, as well as to ensure its excellent water absorption capacity in biomedical applications. 

Furthermore, the controllability of microspheres driven by a rotating magnetic field has also been investigated. Figure 6c shows the timelapse images of a microsphere with a controllable “K” shape at 14 mT and 1 Hz. The microsphere exhibits superior motion controllability in up, down, left, right, and oblique directions, indicating that the developed hydrogel microspheres could achieve controllable and efficient motion, which is expected to be used for targeted drug delivery.

### 2.6. Fluorescence Properties

**Figure 7:**
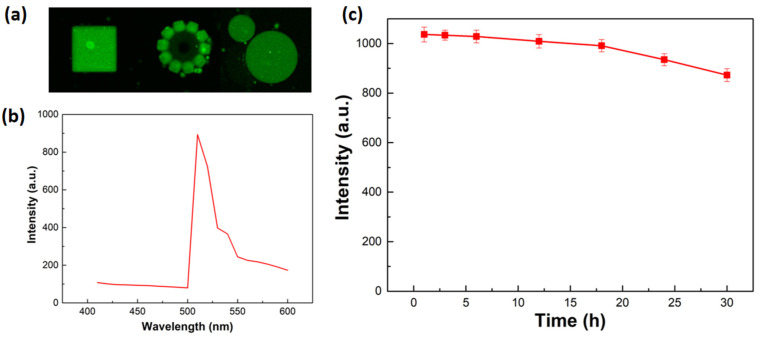
Fluorescence properties of the developed multifunctional hydrogel. (**a**) Fluorescent images of different hydrogel microstructures. (**b**) Fluorescence emission spectra of the hydrogels. (**c**) Fluorescence stability of hydrogels.

Fluorescence-based in vivo imaging has been proven to be a effective tool for monitoring target drug delivery [37]. The developed multifunctional hydrogel has the potential to be used for fluorescence imaging due to its fluorescent molecules. Figure 7a shows the fluorescent images of different hydrogel microstructures under the fluorescence microscope. It can be seen that the microrstructures show good fluorescence properties with clear outlines and uniform fluorescence. Figure 7b shows the fluorescence emission spectra of the hydrogel microstructures measured in DI water at 480 nm excitation, and the fluorescence emission peak was found at 510 nm. Furthermore, the stability of the fluorescent signals was assessed by monitoring the emission intensity against the illumination time. As Figure 7c shows, the intensity of the fluorescent signal slightly decreased from 0 to 30 h and was only reduced by 9.85% after 30 h, which suggests good photostability of the hydrogel. Therefore, the developed multifunctional hydrogel demonstrated stable fluorescence properties, which can fulfill the long-term observation requirements in biomedical applications.

### 2.7. Biodegradability

**Figure 8:**
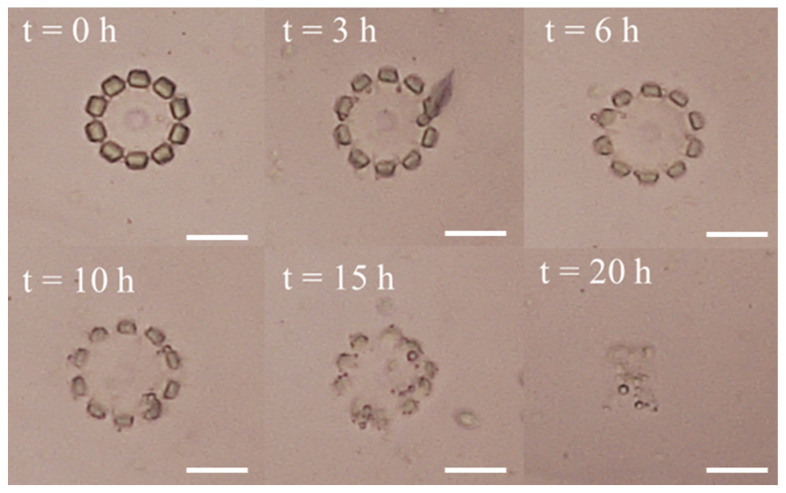
Degradation process of the developed hydrogel petaloid microstructure in collagenase enzyme buffer solution (500 μg/mL). Scale bars: 40 μm.

The monomer of the developed hydrogel is the gelatin methacryloyl (GelMA), which can be degraded by collagenase. This is because the collagenase can break the polypeptide bonds within the gelatin molecules into small molecules and eventually dissolve in solution, as is demonstrated in Figure 1b. In this study, the degradability of developed multifunctional hydrogel was also evaluated. To better understand the degradation process, the petaloid hydrogel microstructures without magnetic nanoparticles were fabricated and then immersed in a 500 μg/mL collagenase enzyme buffer solution, and then were placed in an incubator at 37 °C since collagenase is most active at 37 °C. The petaloid hydrogel microstructures were observed every one hour, and the time-resolved degradation process of the hydrogel microstructure is demonstrated in Figure 8. As shown in this Figure, the hydrogel petaloid microstructure was gradually degraded and then almost completely degraded after 20 h. The results confirmed that the developed multifunctional hydrogel can be degraded by collagenase in 20 h, which indicates that the developed hydrogel has biodegradability and can be degraded in a relatively short time, making it feasible for biomedical applications in vivo.

### 2.8. Biocompatibility

**Figure 9:**
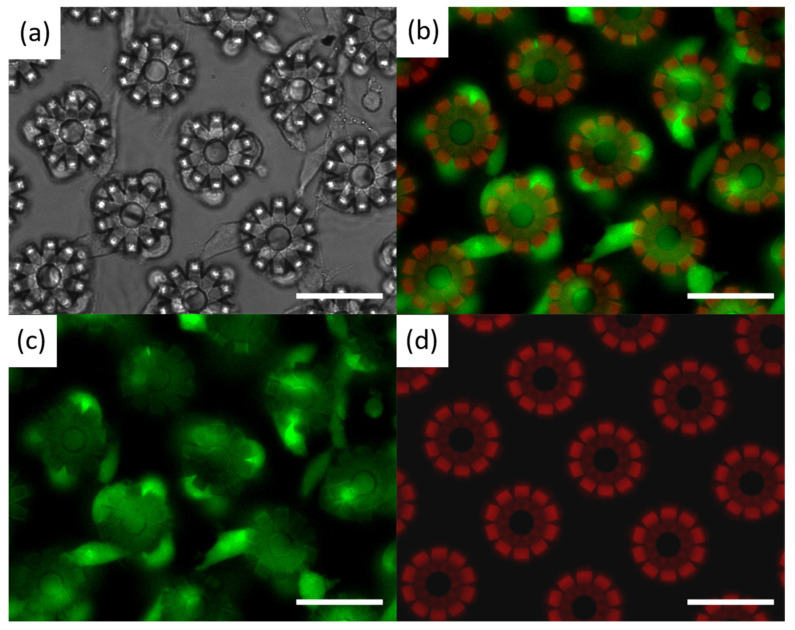
Biocompatibility of developed multifunctional hydrogel. (**a**) Optical image and (**b**) fluorescence image of cultured L929 cells and hydrogel petaloid microstructures. (**c**) Living L929 cells. (**d**) Dead L929 cells and hydrogel petaloid microstructures. The green signals refer to living cells, and the red signals represent hydrogel petaloid microstructures and dead cells. Scale bars: 100 μm.

The biocompatibility of the multifunctional hydrogel was confirmed through in vitro experiments. To demonstrate its efficacy, the L929 fibroblast cells were employed to culture on the printed petaloid microstructures, and the viability of L929 cells was analyzed by observing their morphology with a fluorescence microscope. The images of L929 cells cultured on the printed petaloid microstructures are shown in Figure 8. The optical image in Figure 9a shows that L929 cells grow well on the hydrogel microstructure, indicating that it can serve as dynamic surface that support cell adhesion and growth. Figure 9b–d shows the fluorescence photographs of living cells, dead cells, and hydrogel microstructures under fluorescence microscopy, where the green signals refer to living cells and the red signals represent hydrogel petaloid microstructures and dead cells. The excellent cell viability of the L929 cells can be confirmed from Figure 9c, where living L929 cells were almost fully covered with the entire hydrogel microstructures. Figure 9d further demonstrates the non-cytotoxicity of the developed hydrogel, as dead L929 cells are barely visible on the hydrogel microstructures. Moreover, it is worth mentioning that the fluorescence properties of the developed hydrogel can be further verified in Figure 9d. The results of cell experiments show that the developed multifunctional hydrogel has excellent cytocompatibility and low cytotoxicity, which demonstrates great potential for in vivo biomedical applications. 

## 3. Materials and Methods

### 3.1. Materials

Gelatin methacryloyl (GelMA) (Mw 600, MA degree 97%, Aladdin, Shanghai, China), poly(ethylene glycol) diacrylate (PEGDA) (Mw 600, Sigma-Aldrich, Shanghai, China), triethanolamine (TEOA) (98%, Macklin, Shanghai, China), (2-(4-vinylphenyl)ethene-1,1,2-triyl)tribenzene (TPEMA) (98%, Macklin, Shanghai, China), lithium phenyl-2,4,6-trimethylbenzoylphosphinate (LAP) (Tokyo Chemical Industry Co., Ltd., Tokyo, Japan), and tetrahydrofuran (THF) (Sigma-Aldrich, Shanghai, China).

### 3.2. Synthesis of the Hydrogel Precursor

In a typical procedure, 0.3 g GelMA, 0.03 g LAP, 0.2 mL TEOA, and 0.05 mL PEGDA were added to 0.5 mL PBS buffer solution and then stirred for 1 h (solution A). Afterwards, 10 mg TPEMA was dissolved into 1 mL THF (solution B). Next, 150 μL solution B was added into the former solution A. The hydrogel precursor solution was successfully prepared after being magnetically stirred for 3 h, and then was stored in a UV light-free environment for 3 h before 3D printing. Once made, the precursor was kept in yellow light condition to prevent unnecessary exposure.

### 3.3. Mechanical Property Measurements

The mechanical property of hydrogels was measured using an in situ nanomechanical testing system (FT-NMT03, FemtoTools AG, Buchs, Switzerland). The hydrogel microcuboids with different laser powers were printed and compressed to obtain force-displacement data with distinct curing conditions. 

### 3.4. Fabrication of Fe*_3_*O*_4_*-Coated Microspheres

Before printing, a 22 mm × 22 mm cover glass (Thermo Fisher Scientific Inc., Waltham, MA, USA) was cleaned with isopropyl alcohol (IPA) using ultrasound and then rinsed with deionized (DI) water, followed by nitrogen purging and oxygen plasma treatment. Subsequently, a drop of developed hydrogel precursor solution was placed on the center of the glass substrate using a pipette, and then were loaded in the laser lithography system (Photonic Professional GT, Nanoscribe GmbH, Eggenstein-Leopoldshafen, Germany) and exposed to a laser beam with a center wavelength of 780 nm. The Galvo (layer-by-layer) Scanning Writing Mode was employed to fabricate the sample with high efficiency and precision. After printing, the excess hydrogel precursor solution was removed by developing the printed structures with IPA for 30 min, followed by sample immersion in ultrapure water for at least one hour before testing. Finally, the samples were immersed in ethyl lactate/Fe_3_O_4_ solutions with different concentrations and then by stewing for 12 h to completely evaporate the ethyl lactate. 

### 3.5. Manipulation on the Movement of Microspheres in Magnetic Actuation System

The Fe_3_O_4_-coated microspheres were manipulated in a magnetic actuation system (MFG-100, MagnetbotiX AG, Zurich, Switzerland). The microspheres are immersed in DI water inside a plastic reservoir (30 × 16 × 5 mm) placed at the center of the magnetic actuation system to ensure the uniformity of the magnetic field. Before each experiment, a microprobe (T-4-22, GGB Industries, Inc., Naples, FL, USA) with a sharp tip was used to detach and transport microspheres from the glass substrate onto the smooth Si substrate. Each microsphere was tested at least three times, and the forward/rotational velocity and step-out frequency were measured.

### 3.6. Drug Loading and Release

The fabricated 600 μm × 600 μm × 30 μm hydrogel cube was first dried for 24 h to eliminate water inside the hydrogel, and then was immersed in the urokinase saline solution with a concentration of 5000 u/mL for 24 h to fully absorb the urokinase drug molecules. The hydrogel cube filled with drugs was then removed from the urokinase saline solution and immersed in DI water for 30 s to remove the residual urokinase molecules on the surface of the hydrogel cube. Subsequently, the hydrogel cube were immersed in 10 mL of PBS solution, whereas the release of the embedded urokinase from the hydrogel matrices was monitored for 12 h. Every 10 min, 0.5 mL PBS solution with released urokinase was sampled, examined by UV-Vis spectroscopy, and replaced with fresh PBS solution to approximate perfect sink conditions. Therefore, the accumulated release ϕ is calculated as follows:

$$\phi =\frac{m_{t}}{m_{drug}}\times 100\%=\frac{V_{e}\sum_{1}^{n-1}C_{i}+V_{0}C_{n}}{m_{drug}}\times 100\%$$

where $V_{e}$ is the displacement sampling volume, $V_{0}$ is the initial volume, $C_{i}$ is the concentration of the released urokinase during the i-th displacement sampling, i and n are the sampling times, and $m_{drug}$ is the initial mass of loaded urokinase.

### 3.7. Degradation of GelMA-Based Hydrogel Microstructures

The fabricated petaloid hydrogel microstructures were immersed in a 500 μg/mL collagenase enzyme buffer (Sigma Aldrich), and then were placed in an incubator at 37 °C since collagenase is most active at 37 °C; then, we observed the petaloid hydrogel microstructures every hour.

### 3.8. Fluorescence of Hydrogels

The printed cubic, petaloid, and spherical hydrogel microstructures were observed using a fluorescence microscope (FLS980, Edinburgh Instruments, Livingston, UK), and the intensity of the fluorescent signals was measured every hour.

### 3.9. Cytotoxicity Assay

The L929 fibroblast cells (kindly granted by Prof. Cai Qing, from Beijing University of Chemical Technology) were cultured with complete RPMI medium 1640 (Gibco, Thermo Fisher Scientific Inc., Waltham, MA, USA) containing 10% fetal bovine serum (Gibco, Thermo Fisher Scientific Inc., Waltham, MA, USA) and 1% penicillin-streptomycin solution (Gibco, Thermo Fisher Scientific Inc., Waltham, MA, USA) in a 5% CO_2_ incubator at 37 °C. Before cell culturing, the printed hydrogel microstructures were soaked in water for 24 h and changed the water every 3 h. Cells were treated with trypsin-EDTA (Gibco, Thermo Fisher Scientific Inc., Waltham, MA, USA) and resuspended with complete RPMI medium 1640. Subsequently, the cells with a density of 1 × 10^5^ were seeded onto the printed hydrogel microstructures and allowed to grow for 24 h. The cytocompatibility of the hydrogels was analyzed using a Live/Dead assay. In total, 22 μM calcein AM (in DPBS) and 4 μM EthD-1 (Invitrogen, Thermo Fisher Scientific Inc., Waltham, MA, USA) working solution were added to the Petri dish after washing the dish with DPBS (Gibco, Thermo Fisher Scientific Inc., Waltham, MA, USA). Afterwards, the Petri dish was then incubated in a 5% CO_2_ incubator at 37 °C for 20 min. A Fluorescence microscope (Olympus IX71, Tokyo, Japan) was employed to observe the morphologies of the cells.

## 4. Conclusions

In this paper, a multifunctional hydrogel with 3D printability, fluorescence imageability, degradability, and biocompatibility was developed. The hydrogel is made up of GelMA (a monomer), TPEMA (a fluorescent agent), PEGDA (a crosslinker), TEOA (a photosensitizer), and LAP (a photoinitiator). The multifunctional hydrogel exhibits excellent fluorescence stability, with the fluorescent signal only decreasing by 9.85% after 30 h. The hydrogel microstructures can be degraded by collagenase and almost completely degraded after 20 h. Additionally, the hydrogel also demonstrates excellent cytocompatibility and low cytotoxicity. The L929 cells survive well on the hydrogel microstructure, making it useful as a scaffold for cell growth and adhesion. Furthermore, Fe_3_O_4_-coated microspheres have been developed based on the multifunctional hydrogel, which demonstrate controllable motion and excellent water absorption capacity, making them useful for targeted delivery of water-soluble drugs. The proposed hydrogel and microspheres together are a promising candidate for real in vivo biomedical applications where 3D printable, motion-controllable, imageable, degradable, and biocompatible microrobots are needed simultaneously.

## Data Availability

Data are contained within the article.
